# Potential serum biomarkers and metabonomic profiling of serum in ischemic stroke patients using UPLC/Q-TOF MS/MS

**DOI:** 10.1371/journal.pone.0189009

**Published:** 2017-12-11

**Authors:** Hongxue Sun, Jiaying Zhao, Di Zhong, Guozhong Li

**Affiliations:** 1 Department of Neurology, The First Affiliated Hospital of Harbin Medical University, Harbin, Heilong Jiang Province, PR China; 2 Department of Thoracic Surgery, The Second Affiliated Hospital of Harbin Medical University, Harbin, Heilongjiang, China; Georgetown University, UNITED STATES

## Abstract

**Background:**

Stroke still has a high incidence with a tremendous public health burden and it is a leading cause of mortality and disability. However, biomarkers for early diagnosis are absent and the metabolic alterations associated with ischemic stroke are not clearly understood. The objectives of this case-control study are to identify serum biomarkers and explore the metabolic alterations of ischemic stroke.

**Methods:**

Metabonomic analysis was performed using ultra-performance liquid chromatography quadrupole time-of-flight tandem mass spectrometry and multivariate statistical analysis was employed to study 60 patients with or without ischemic stroke (30 cases and 30 controls).

**Results:**

Serum metabolic profiling identified a series of 12 metabolites with significant alterations, and the related metabolic pathways involved glycerophospholipid, sphingolipid, phospholipid, fat acid, acylcarnitine, heme, and purine metabolism. Subsequently, multiple logistic regression analyses of these metabolites showed uric acid, sphinganine and adrenoyl ethanolamide were potential biomarkers of ischemic stroke with an area under the receiver operating characteristic curve of 0.941.

**Conclusions:**

These findings provide insights into the early diagnosis and potential pathophysiology of ischemic stroke.

## Introduction

Ischemic stroke (IS) occurs when the blood supply to the brain is interrupted, usually when a blood vessel bursts or is blocked by a clot and the supply of oxygen and nutrients are cut off, causing damage to the brain tissue [1]. Some preventive measures aimed at high-risk factors could be implemented, including changes in diet, lifestyle modification, and drug intervention. However, the incidence of stroke remains high, especially in developing countries, with tremendous medical costs [2]. In 2013, approximately 6.9 million people worldwide had an IS and, in China, it was approximately one million and a leading cause of mortality and disability [3, 4]. If an IS can be detected within three to four and half hours, the clot may be broken down with medication [5]. Therefore, IS patients should be evaluated rapidly for an early diagnosis and considered for timely symptomatic treatment. Typical diagnosis is with medical imaging, such as a computed tomography (CT) or magnetic resonance imaging (MRI) scan along with a physical exam, and other tests of electrocardiogram and blood tests may also be done to rule out other possible causes [6]. However, these examinations are time-consuming and expensive. Therefore, developing novel biomarkers through metabonomics may improve the clinical diagnosis of IS and timely clinical treatment as well as find potential metabolic changes and mechanisms by exploring the pathways associated with IS.

Metabonomics is an unbiased analytical method to obtain a quantitative measurement of the metabolic response of whole organisms to pathophysiological stimuli [7], which has been applied widely for biomarker discovery [8, 9] and to elucidate the pathogenesis of diseases [10, 11]. However, few metabonomic studies of stroke have been conducted using different techniques in various biological samples [12, 13]. A plasma and urine study using a nuclear magnetic resonance (NMR) spectroscopy method in patients with cerebral infarction has shown that the cerebral infarctions were associated with anaerobic glycolysis, folic acid deficiency, and hyperhomocysteinemia [12]. Another targeted metabonomics study using liquid chromatography followed by tandem mass spectrometry, and the findings suggested that plasma branched-chain amino acids (valine, leucine, and isoleucine) could be diagnostic markers for IS [13]. However, the targeted metabonomics approach only determines a set of metabolites rather than many metabolites as possible base on the sensitivity and resolution of instruments in a non-targeted metabonomics approach. This approach limited the collection of biomarkers and found of important metabolic changes of disease. In addition, compared with other analytical technologies, ultra-performance liquid chromatography (UPLC) coupled with mass spectrometry (MS) has been applied widely in metabonomic studies owing to its higher resolution, sensitivity and wider detection of metabolites [14, 15].

In the present study, a serum metabonomic analysis was made usingultra-performance liquid chromatography quadrupole time-of-flight tandem mass spectrometry (UPLC/Q-TOF MS/MS) to identify potential serum biomarkers and explore the metabolic alterations associated with IS in a cohort of patients with IS and healthy individuals.

## Subjects and methods

### Subjects

Sixty subjects, 41 males and 19 females, 42 to 79 years of age, were recruited at the First Affiliated Hospital of Harbin Medical University from March 2017 to June 2017. All patients (n = 30) were confirmed by CT or MRI scans of the brain according to the evaluation of National Institutes of Health and Stroke Scale (NIHSS) at admission [16]. Age- and sex-matched volunteers and healthy individuals as control subjects (n = 30) were recruited in the general outpatient department according to an assessment by questionnaire for verifying stroke-free status. Patients with a history of autoimmune disease, pregnancy, serious brain disorders, regular immunosuppressive or analgesic therapies, and concurrent cancerous disease or liver or kidney failure were excluded from the study. Written informed consent was obtained from all participants, and the study protocol was approved by the Ethics Committee of the First Affiliated Hospital of Harbin Medical University (NO.201726) and conducted in accordance with the Declaration of Helsinki. The study was registered at Chinese Clinical Trial Registry, and the registration number was ChiCTR-BOC-17011771.

### Clinical examination

Data were collected on age, alcohol intake, smoking, and health history. Weight was measured twice (with an accuracy of ± 0.1 kg) after fasting overnight and wearing only underwear. Blood pressure was measured on the right arm by a mercury sphygmomanometer (with at least 10 minutes of rest). After overnight fasting of 12 hours, venous blood was collected, centrifuged at 3 000 × g for 15 min, and serum was extracted and stored at –80°C for sample analysis. Serum total cholesterol (TC), triglycerides (TG) and high-density/low-density lipoprotein cholesterol (LDL-C/ HDL-C) were determined by commercial kits (Biosino Biotechnology Ltd, Beijing, China). A Kyoto blood sugar test meter and test strip (Arkray, Inc. Kyoto, Japan) were used to determine the level of plasma glucose, and the level of high-sensitivity C-reactive protein (hs-CRP) was determined using an enzyme immunoassay assay kit (Quantikine Human C-Reactive Protein Immunoassay, R&D Systems Inc., Minneapolis, MN, USA).

### Chemicals

Chromatography-grade acetonitrile and methanol were purchased from Honeywell, Burdick, & Jackson (Muskegon, MI, USA). Leucine enkephalin and analytical grade formic acid were purchased from Sigma-Aldrich (St Louis, MO, USA) and Harbin Biotechnology Company (Harbin, China), respectively. Standards were obtained from commercial sources.

### Global serum metabolic profiling analysis using UPLC-Q-TOF-MS/MS

#### Sample preparation

Samples were prepared as described previously [17, 18]. After deproteinization with methanol (1:5), the supernatant of serum samples was dried with nitrogen at 37°C, dissolved in acetonitrile and water (3:1) and centrifuged at 10 000 × g for 15 min. The supernatant was transferred into autosampler vials. To verify the reproducibility and reliability of data, a typical pooled quality control (QC) sample was prepared by mixing equal volumes of serum samples from six healthy subjects and six stroke patients and injected every 15th samples intermittently throughout the run. To avoid the order effects in statistical analysis, a randomized crossover design was used. The samples in two batches of analysis were injected alternately between five healthy subjects and five stroke patients.

#### UPLC-QTOF-MS/MS conditions

UPLC-Q-TOF-MS/MS analysis was performed by an Acquity UPLC system coupled to a quadrupole time-of-flight tandem mass spectrometer with electrospray ionization (ESI) in positive mode (Waters Corporation). A 2-μL sample was injected into aBEH-C_18_ column (4.6 ×50 mm, 1.7 μm; Waters) using an Acquity UPLC system. The mobile phase consisted of two solutions including solvent A (0.1% formic acid in water) and solvent B (acetonitrile), and the flow rate was 0.3 mL/min. The initial composition of B was 2% and increased to 20% from 0–1.5 min, 20–70% from 1.5–6 min, and 70–92% from 6–9 min, 92–98% from 9–15 min, 98–98% from 15–16.5 min, followed by re-equilibration to the initial conditions in 4 min. For MS analysis in positive ESI mode, the capillary voltage and sample cone voltage were set at 3000 V and 35 V, respectively. The desolvation temperature was 300°C and the gas flow was 600 L/h. The source temperature was set at 100°C and the cone gas flow was 50 L/h. The centroid data were collected from 80−1000 m/z with an acquisition rate of 0.4 s and a 0.1 s interscan delay. For ensuring accuracy and reproducibility, leucine enkephalin in 200 pg/mL was used as the lock-mass for positive ESI mode ([M+H]+ = 556.2771) via a lock spray interface.

#### Data processing

Data were processed as described previously [17, 18]. The raw data were imported into the MarkerLynx Application Manager 4.1 SCN 714 (Waters Corporation, Milford, MA, USA) for peak detection and alignment, and the collection of parameters were set as follows: noise elimination level 10.00, mass window 0.05 Da, and retention time tolerance 0.01 min. Data were only used up to 16.5 minutes after the point where the column-washing phase started. The resulting matrix, including sample name, retention times, m/z, and normalized peak areas, were exported to EZinfo software (version 2.0.0.0, Waters) for further multivariate statistical analysis. The unsupervised principal components analysis (PCA) can reduce the dimensionalities of complex datasets and provide an overview of all observations. PCA was used to visualize the initial overview of quality of the analytical run for the data quality assessment of the metabonomics platform. Supervised partial least squares-discriminant analysis (PLS-DA) was applied to visualize the maximal difference between two groups. The independent samples t-test was applied to measure the significance of each metabolite. Potential biomarkers were selected according to the greatest variable importance in projection (VIP) values and statistical significance (VIP > 1.5 and P < 0.05). The goodness of the fit was quantified by R2Y, while the predictive ability was indicated by Q2, and models with R2Y and Q2 greater than or equal to 0.5 were considered to be suitable for the recognition analysis [19]. For avoiding the over-fitting of supervised PLS-DA models, a cross-validation procedure and testing with 200 random permutations were performed using SIMCA-P software (version 11.5; Umetrics AB, Umeå, Sweden).

The probable empirical formulas of the potential biomarkers were first identified based on accurate mass measurement and the relative intensities of the isotope peaks through the high-resolution MS spectra. Then, the MassFragment application manager was used to facilitate the MS/MS fragment ion-analysis process by chemically intelligent peak-matching algorithms. Briefly, the exact mass and MS/MS spectra of metabolites were matched with the structure details of metabolites obtained from the Human Metabolome Database (HMDB, http://www.hmdb.ca/) according to the parameters including deviation from calculated mass, isotopic pattern, and double bond equivalent. Finally, the metabolites were confirmed by standard compounds based on both retention times and MS/MS spectra. The pathway analysis of potential biomarkers was first performed with MetaboAnalyst 3.0 (http://www.metaboanalyst.ca/), which was based on database sources including KEGG (http://www.genome.jp/kegg/) and HMDB to identify the top altered pathways analysis and visualization [20]. Then, other implicated pathways of biomarkers were further interpreted using references and databases.

### Statistical analysis

Statistical analysis was performed using SPSS (version 13.01S; Beijing Stats Data Mining Co. Ltd., Beijing, China). Data were presented as the mean ± S.D. or the percentage, as appropriate. Differences between groups were analyzed by independent samples *t*-test. The chi-squared test was used to compare categorical variables. Multiple logistic regression analysis of top potential metabolites was performed using stepwise selection. Receiver operating characteristics (ROC) analysis was used to evaluate predictive ability of potential metabolic biomarkers, area under the curve (AUC), and sensitivity and specificity were determined according to the maximum value of the Youden index. P values are 2-tailed and P < 0.05 was considered statistically significant.

## Results

### Clinical characteristics

There were no significant differences in age, sex, TG, and HDL-C between healthy and stroke groups. The systolic blood pressure, diastolic blood pressure, TC, LDL-C, fasting plasma glucose, and Hs-CRP were significantly higher in stroke patients than in healthy subjects **(Table 1)**.

**Table 1:** Mean ± SD of study variables in control and ischemic stroke groups.

	Control	Ischemic stroke	*P* value
**Age (years)**	57.83 ± 11.08	59.77 ± 12.41	0.527
**Male Sex (%)**	60.0	53.3	0.602
**Weight (kg)**	65.33 ± 11.09	68.83 ± 8.76	0.180
**SBP (mmHg)**	116.9 ± 12.01	137.37 ± 21.46	< 0.001
**DBP (mm Hg)**	76.63 ± 6.97	84.77 ± 14.27	0.007
**TG (mmol/L)**	4.5 ± 0.84	5.14 ± 0.83	0.065
**TC (mmol/L)**	1.44 ± 0.55	1.76 ± 0.76	0.004
**HDL-C**	1.36 ± 0.4	1.23 ± 0.37	0.218
**LDL-C**	2.79 ± 0.56	3.69 ± 0.72	< 0.001
**FPG (mmol/L)**	5.75 ± 0.88	6.63 ± 1.23	0.002
**Hs-CRP (mg /L)**	3.73 ± 1.17	8.32 ± 1.97	< 0.001

SBP = systolic blood pressure; DBP = diastolic blood pressure; FPG = fasting plasma glucose; TC = total cholesterol; TG = triglycerides; Hs-CRP = high-sensitivity C-reactive protein.

### Data quality assessment of the metabonomics platform

**Fig 1:**
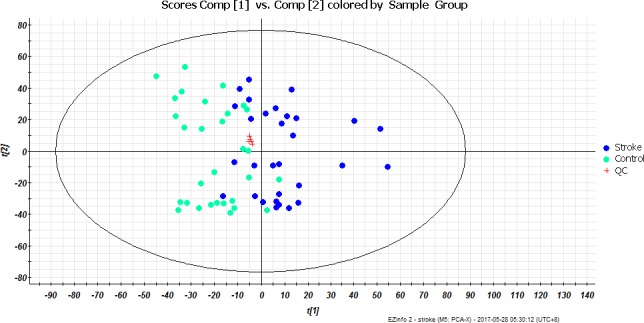
Two-dimensional principal components analysis (PCA) score plots of stroke and control groups in the positive mode. t[1] = first principal component; t[2] = second principal component. The QC samples were tightly clustered in the PCA score plot and showing minimal analytical variation.

An initial overview of the quality of the analytical run was obtained by PCA on the sample dataset included QC injections. The results showed that the QC samples were clustered tightly in the scores plot (**Fig 1**: Red Cross). The relative standard deviations (RSDs) of retention time ranged from 0.07% to 0.91%, and the RSDs of peak intensity ranged from 1.21% to 5.17% in positive mode (**S1 Table**). The results showed that the precision and repeatability of the experiments were excellent, which was sufficient to ensure the data quality of the global metabonomics analysis.

### Serum metabolic profiling analysis of healthy and stroke subjects

**Fig 2:**
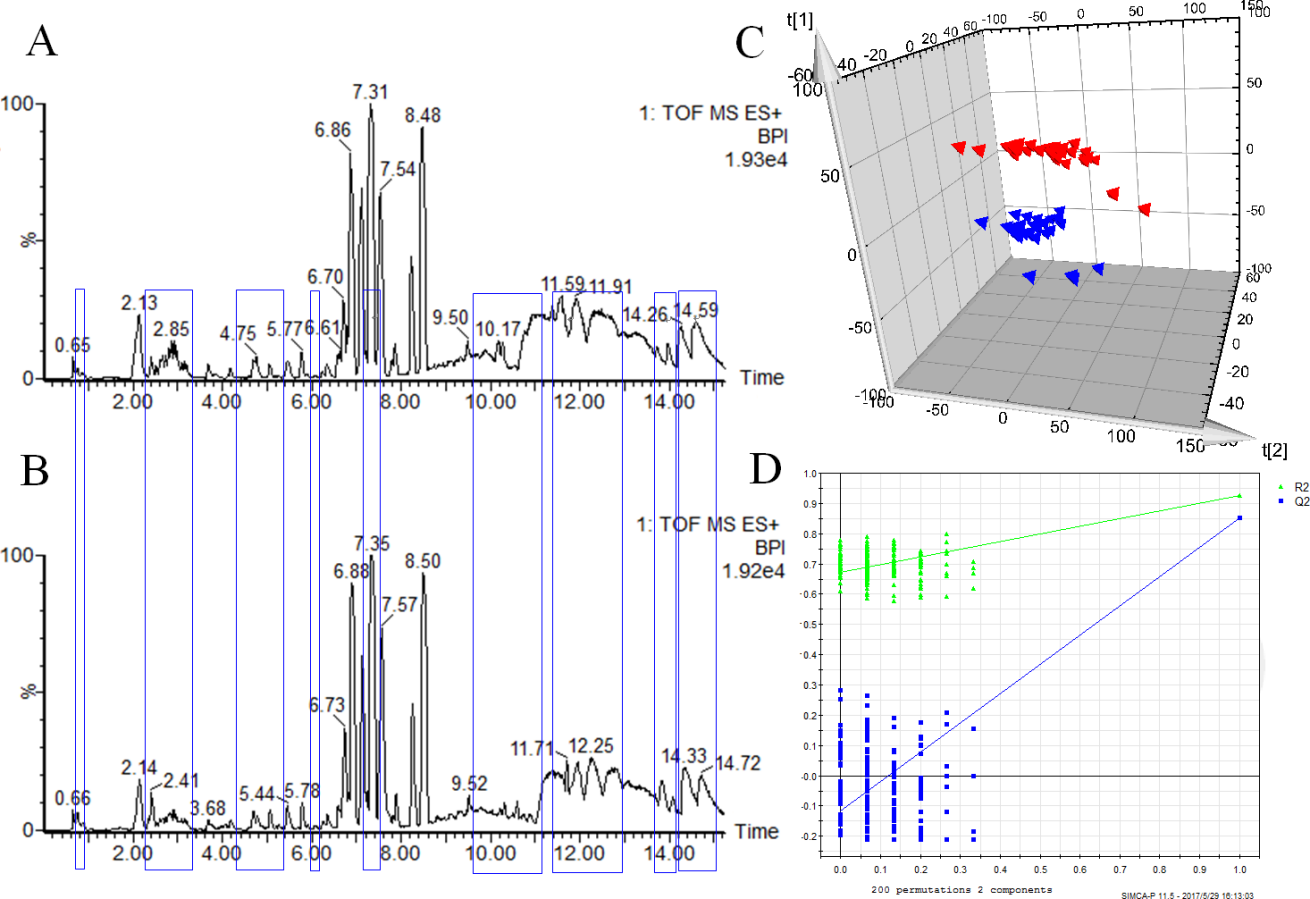
Serum metabolic profiling and score plot with PLS-DA. A and B: metabolic profiling in control and stroke groups, respectively. Blue box marked the differences of high or area between the two chromatograms; C: the controls are indicated by triangles and stroke patients by blue triangles. Each data point represents one subject. Comp, component. t[1], component 1; t[2], component 2; One data point represents one subject; D: permutation test result of the PLS-DA model in the positive ESI mode. The R2Y value represents the goodness of fit of the model. The Q2 value represents the predictability of the models. R2Y (green triangle) = 0.927 and Q2 (blue box) = 0.853. All R2Y and Q2 values to the left were lower than the original points to the right, showing that the PLS-DA model was valid.

The typical based peak intensity (BPI) chromatograms of serum samples of the two groups are shown in **Fig 2A and 2B**. The data (3949 variables) were used for multivariate statistical analysis. As shown by the PLS-DA scores plot (**Fig 2C**), according to the metabolic differences, healthy and stroke subjects could be separated into distinct clusters (PLS-DA models: R2Y = 0.927 and Q2 = 0.853). All values were more than 0.5, which indicates that the models were suitable for recognition analyses. In addition, the permutation test for PLS-DA showed that the Q2 regression line had a negative intercept and all R^2^and Q^2^ values on the left were lower than the original points on the right **(Fig 2D)**, which showed that the PLS-DA model in the present study was valid. In total, according to the parameter variable importance in the projection, 12 different metabolites with important roles in the separation were selected and were identified **(Table 2 and S1 Fig)**. The mass fragment information of metabolites, used for the identification of metabolites, is shown in **S2 Fig**.

**Table 2:** Identification and changing trends for the principal metabolites of ischemic stroke.

Retention time (min)	Measured mass (Da)	Calculated mass	Mass error(PPM)	Elemental composition		Identity	VIP	Fold change *	*P*
0.89	169.0355	169.0362	4.14	C5H4N4O3		Uric acid ^a^	3.42	1.73	<0.001
2.76	476.2685	476.2777	19.32	C23H42NO7P		LysoPE(0:0/18:3) ^a^	2.77	0.60	0.002
2.81	520.3399	520.3403	0.77	C26H50NO7P		LysoPC(18:2) ^a^	3.76	0.62	<0.001
4.72	585.2626	585.2713	14.87	C33H36N4O6		Bilirubin ^a^	4.82	0.05	<0.001
6.23	302.3072	302.3059	4.30	C18H39NO2		Sphinganine ^a^	3.69	1.66	<0.001
6.98	424.3454	424.3427	6.36	C25H45NO4		Linoelaidyl carnitine ^b^	4.55	2.04	<0.001
7.41	496.3365	496.3403	7.66	C24H50NO7P		LysoPC(16:0) ^a^	8.62	0.83	<0.001
9.58	376.3187	376.3216	7.71	C24H41NO2		Adrenoyl ethanolamide ^b^	3.10	1.81	<0.001
12.09	760.5815	760.5856	5.39	C42H82NO8P		PE(15:0/22:1) ^a^	14.44	2.86	<0.001
13.06	778.5464	778.5387	9.89	C42H68NO10P		PS(14:1/22:6)^b^	3.38	0.15	<0.001
13.70	754.5444	754.5387	7.55	C42H76NO8P		PC(14:0/20:4) ^a^	2.11	0.45	<0.001
14.93	806.5831	806.5700	16.24	C46H80NO8P		PC(16:0/22:6) ^a^	11.22	0.56	0.001

* Ratio of mean relative amount between the stroke and control groups.

a: Metabolites were identified with exact mass data, MS fragmentation, retention time and confirmed using a standard, and

b: Metabolites were identified by exact mass data, MS fragmentation, and retention time.

### Multiple logistic regression analysis

**Fig 3:**
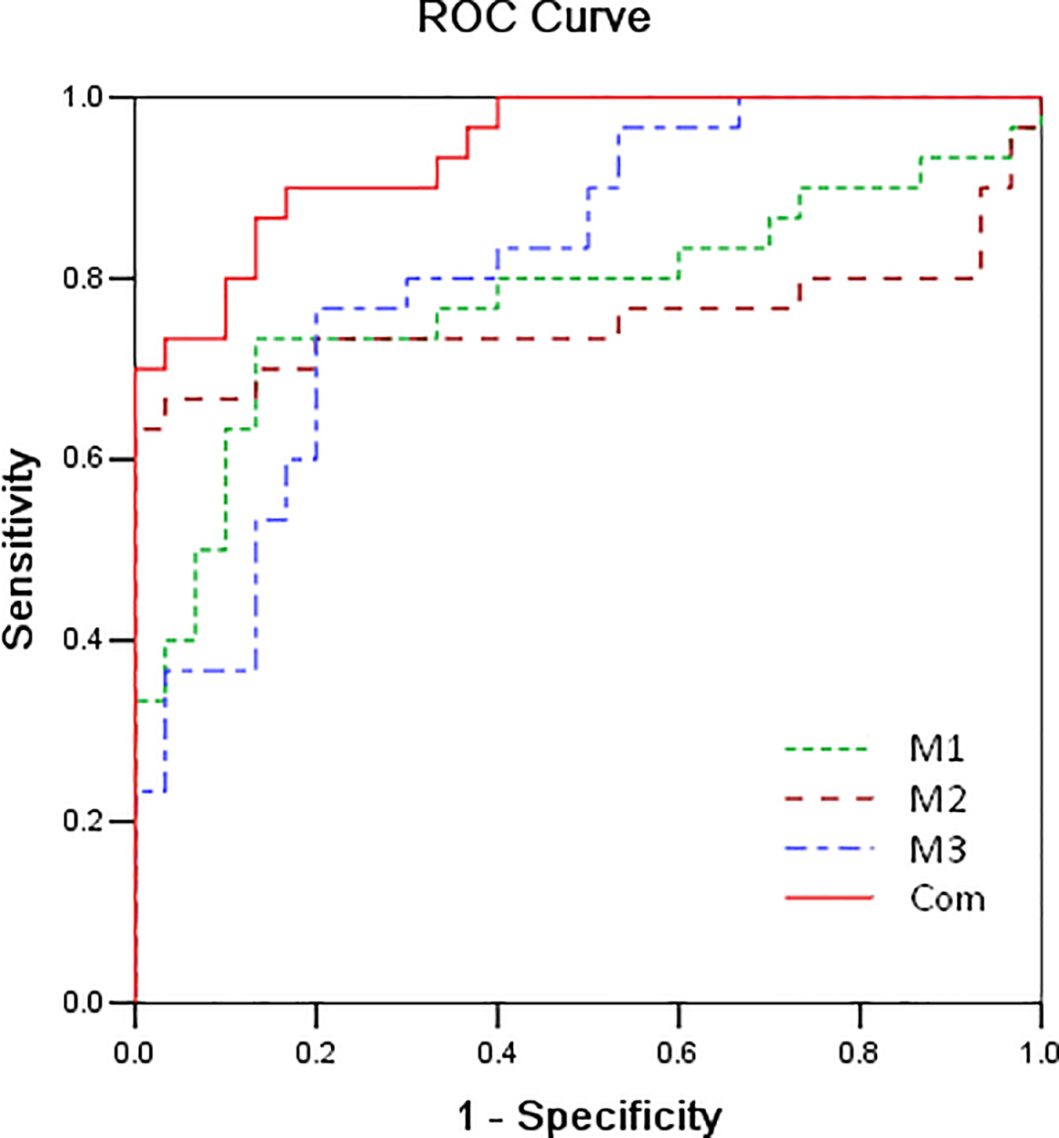
Areas under ROC curve of the biomarkers and combination of the three biomarkers. Marker 1, uric acid; marker 2, sphinganine; marker 3, adrenoyl ethanolamide. Red line combination of three biomarkers: AUC = 0.941.

To assess how multiple metabolites collectively classify the disease status of stroke, we built a multiple regression model using stepwise selection. The regression model was first built on 12 metabolites, controlling for age and sex. Three metabolites, uric acid (β = 0.194, p = 0.001), sphinganine (β = 0.186, p = 0.005), and adrenoyl ethanolamide (β = 0.177, p = 0.027), included in the multiple regression model, explained 72.4% of the variation. We calculated the sensitivity and specificity based on estimates of the final model, and the model fits very well (AUC = 0.941, sensitivity = 90%, specificity = 83%; **Fig 3**). Compared with the AUC of simple metabolites in **Table 3 and Fig 3**, thus, the combination of uric acid, sphinganine, and adrenoyl ethanolamide was selected as the candidate biomarkers with highest AUC for diagnosis of IS.

**Table 3:** Receiver operator characteristic curve analysis of 12 metabolites.

Biomarkers	AUC	Sensitivity (%)	Specificity (%)	Maximum of Youden index*
**Uric acid**	0.778	73	77	0.50
**LysoPE(0:0/18:3)**	0.730	77	67	0.43
**LysoPC(18:2)**	0.748	67	73	0.40
**Bilirubin**	0.859	80	73	0.53
**Sphinganine**	0.754	70	83	0.53
**Linoelaidyl carnitine**	0.903	77	87	0.63
**LysoPC(16:0)**	0.794	63	83	0.47
**Adrenoyl ethanolamide**	0.814	77	77	0.53
**PE(15:0/22:1)**	0.903	83	87	0.70
**PS(14:1/22:6)**	0.894	80	73	0.53
**PC(14:0/20:4)**	0.787	73	83	0.57
**PC(16:0/22:6)**	0.732	60	80	0.40

AUC = area under the curve

* Sensitivity + specificity– 1.

### Metabolic pathways

**Fig 4:**
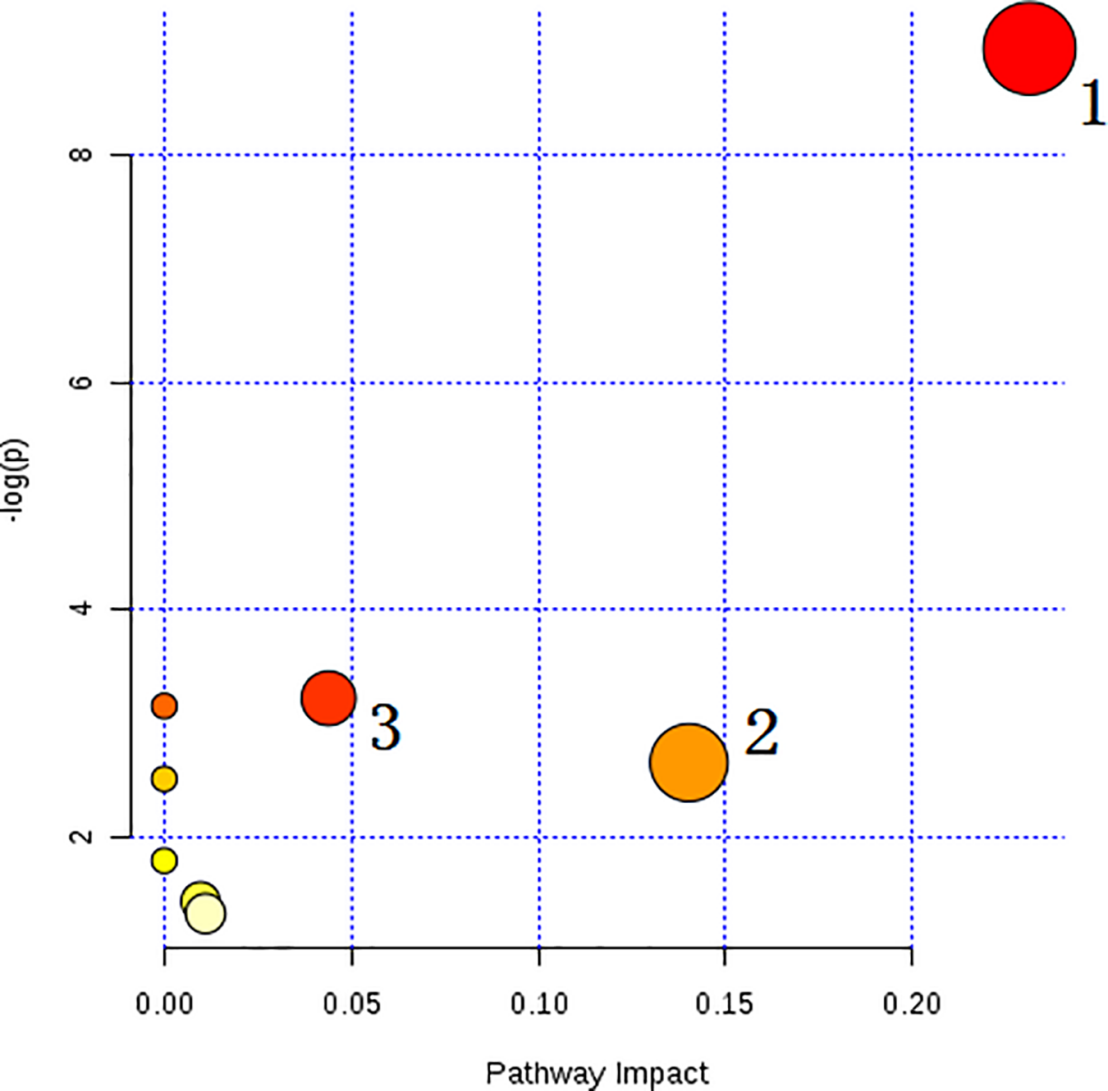
Summary of pathway analysis for biomarkers in MetaboAnalyst3.0. (1) Glycerophospholipid metabolism; (2) Sphingolipid metabolism; (3) Glycosylphosphatidylinositol (GPI)-anchor biosynthesis.

The potential target metabolic pathway analysis (impact value ≥ 0.10) with MetaboAnalyst 3.0 revealed that two metabolic pathways, glycerophospholipid metabolism and sphingolipid metabolism, were found to be associated with IS (**Fig 4**).

## Discussion

We performed a metabonomic analysis on serum from a cohort of patients with IS and controls and identified 12 metabolites that may enable the classification of disease status. Multiple logistic regression analysis of these 12 metabolites showed that three of the metabolites identified, uric acid, sphinganine, and adrenoyl ethanolamide, were significant biomarkers of IS. Through pathway analysis of these principle metabolites, the important metabolic changes of IS were found. Finding biomarkers and the metabolic changes of IS could provide information for a rapid evaluation and an early diagnosis to target therapy.

Through the potential target metabolic pathway analysis with MetaboAnalyst 3.0, two metabolic pathways, glycerophospholipid metabolism and sphingolipid metabolism, were found to be associated with IS. Phosphatidylethanolamine (PE), phosphatidylcholine (PC), and LysoPC are the intermediates in glycerophospholipid metabolism. It has been reported previously [21] that glycerophospholipid constitutes the backbone of the neural membrane, which provides a suitable environment, fluidity, and ion permeability. Brain polyunsaturated fatty acids (PUFAs), such as docosahexaenoic acid and arachidonic acid, can be released from the degradation of glycerophospholipids. Several lipid mediators can be produced by oxidation of these PUFAs, which are all closely related with neuronal pathways involved in neurodegenerative diseases, and also suggests that an interplay among lipids occurs in brain tissue. The results suggest that the alteration of glycerophospholipid metabolism could interpret the reason for the cerebral damage from IS. Sphinganine is an intermediate in sphingolipid metabolism. Sphingolipids are a series of cell membrane-derived lipids which act as signaling molecules and play a critical role in cell death and survival, proliferation, recognition, and migration. It has been reported that sphingolipid activities change after stroke and correlate with stroke outcome, and the metabolic pathway of sphingolipid was closely correlated with stroke, which maybe a novel therapeutic target in stroke for reducing stroke-induced brain injury [22].

Metabolic pathways analysis was also carried out by references and databases. Abnormal phospholipid metabolism that correlated with lysophosphatidylethanolamine (LysoPE) and phosphatidylserine (PS) were observed. Two biomarkers found in phospholipids were correlated with IS. LysoPE is a hydrolysis product of PE by phospholipase A2, which plays a role in cell-mediated cell signaling and activation of other enzymes [23]. PS is a glycerophospholipid in which a phosphorylserine moiety occupies a glycerol substitution site. Previous studies have reported abnormal levels of phospholipid in serum [24, 25]. In addition, recent structural and functional data identify phospholipids (such as PS) as a major bioactive component of high-density lipoproteins (HDLs) [26]. HDLs play a major protective role in acute stroke due to their antioxidant, anti-inflammatory, and antithrombotic properties, in particular, by limiting the deleterious effects of ischemia on the blood–brain barrier and on the parenchymal cerebral compartment [27]. In the present study, the significantly decreased LysoPE and PS in the serum of patients with IS suggest an abnormity of phospholipid metabolism in patients with IS, and also maybe one of the potential reasons for IS. Abnormal fatty acid metabolism that correlated with adrenoyl ethanolamide was also observed. Adrenoyl ethanolamide is an N-acylethanolamine (NAE). NAEs are derivatives of fatty acids in which the carboxylic group of the fatty acid is bound by an amide linkage to the amino group of ethanolamine [28]. Prior studies have demonstrated that NAEs have neurotrophic/neuroprotective activities through a broad spectrum of cellular and animal models of neurodegenerative and acute cerebrovascular disorders [29]. NAEs are signaling lipids whose synthesis is upregulated in response to ischemia, and serum NAE concentration increased about 30-fold in a rat study after stroke [30]. The increase of adrenoyl ethanolamide in our study may be a self-protective mechanism during cerebral damage caused by IS.

We also found abnormal acylcarnitine metabolism. Linoelaidyl carnitine is along-chain acylcarnitine. Makrecka *et al*. [31] showed that long-chain acylcarnitine could regulate the metabolism of pyruvate–lactate and long-chain fatty acids in the mitochondria, and the increase of long-chain acylcarnitine could impair glucose metabolism. A study by Guasch-Ferré *et al*. also indicated that the elevated concentrations of acylcarnitines are independently associated with risk of stroke [32]. Therefore, the increase of linoelaidyl carnitine may be one of the causes of high levels of serum glucose in patients with IS in the present study, which also might contribute to the occurrence of IS.

Other abnormal metabolic pathways found in the present study were correlated with biomarkers of bilirubin and uric acid. Bilirubin is an end product of heme metabolism and has been reported to have powerful antioxidant and anti-inflammatory effects properties [33, 34], and there was an inverse relationship between serum bilirubin and CRP [34, 35]. Moreover, Kimm *et al*. found that serum bilirubin has a protective function against stroke risk, and a low serum bilirubin level could be an independent predictor of stroke incidence [36]. Therefore, the low level of bilirubin in patients might partly explain the reasons for the high levels of CRP and the occurrence of IS in the study. Uric acid, as a product of purine metabolism, is a powerful endogenous antioxidant which may increase in many oxidative stress situations such as hypertension [37]. Hypertension is a major risk factor for cerebrovascular disease. A meta-analysis of 18 cohort studies showed that hyperuricemia was associated with an increased risk in the incidence of hypertension [38]. In addition, Hozawa *et al*. found that uric acid is an independent predictor of IS [39]. Uric acid is biologically active and can stimulate oxidative stress, endothelial dysfunction, inflammation and vasoconstriction [40]. The level of serum uric acid is closely related to the occurrence of stroke. Increased serum uric acid may increase the risk of thrombosis by promoting lipid peroxidation, the oxidation of low-density lipoprotein, an increase of oxygen free radicals and platelet adhesion, and promote platelet thrombus formation in early thrombosis. Moreover, Uric acid crystallization can deposit in the vessel wall, to injure the endothelium directly, induce inflammation, and promote the formation of atherosclerosis. Atherosclerosis is the most important factor and link in the mechanism of stroke and the determination of serum uric acid levels can be used to speculate the risk of IS [37,40,41]. Our study showed higher levels of blood pressure in patients with IS, which may be from high serum uric acid and together they could contribute to the occurrence of IS.

In the present study, some metabolic changes were investigated through pathway analysis of identified biomarkers. However, some principle metabolites remain unidentified at present, which maybe a possible limitation of this study. These unidentified metabolites and their metabolic pathways might include other important metabolic changes. In addition, the sample size was relatively small. Further validation with a larger sample or with a longitudinal study on our findings may need to be done to control for intraindividual variations. We will devote our future research to improving the capacity of metabolite identification and related metabonomic study in stroke.

## Conclusion

In summary, we characterized, for the first time, the altered metabolites by a distinct serum metabolic profiling and identified 12 biomarkers related to IS, and three metabolites, uric acid, sphinganine, and adrenoyl ethanolamide, were identified as the potential biomarkers of IS, which may have clinical applications for IS. Our study may also provide a novel insight into the pathophysiological changes and pathogenesis of IS.

## Supporting information

S1 Table Repeatability of the experimental method from six ions of the quality control sample. (DOC)

S1 Fig Trending plot of 12 metabolites in positive ESI mode. (DOC)

S2 Fig Mass fragment information of principal metabolites in positive ion mode. (DOC)
