# Supplementary material for: Potential serum biomarkers and metabonomic profiling of serum in ischemic stroke patients using UPLC/Q-TOF MS/MS
Source: PLoS One. 2017 Dec 11;12(12):e0189009. doi: 10.1371/journal.pone.0189009 (PMC5724857; doi:10.1371/journal.pone.0189009)
Supplement: S1 Table — (DOC) [file pone.0189009.s001.doc]

**Table S1. Repeatability of experimental method from six ions of the quality control sample (n = 5).**

| **m/z** | **RT (min)** | | |  | **INT(10^3^)** | | |
| --- | --- | --- | --- | --- | --- | --- | --- |
|  | **Mean** | **SD** | **RSD (%)** |  | **Mean (10^2^)** | **SD (10^2^)** | **RSD (%)** |
| 100.0726 | 2.16 | 0.008 | 0.37 |  | 4.17 | 0.05 | 1.29 |
| 226.9527 | 0.66 | 0.006 | 0.91 |  | 0.53 | 0.01 | 1.21 |
| 274.2734 | 5.45 | 0.006 | 0.11 |  | 2.23 | 0.06 | 2.83 |
| 338.3407 | 11.78 | 0.005 | 0.04 |  | 2.93 | 0.15 | 5.17 |
| 496.3044 | 7.37 | 0.013 | 0.18 |  | 17.07 | 0.62 | 3.61 |
| 991.6746 | 7.12 | 0.005 | 0.07 |  | 2.22 | 0.10 | 4.60 |

RT: retention time; INT: intensity; SD: standard deviation; RSD: relative standard deviation
