# Supplementary figures and images for: Potential serum biomarkers and metabonomic profiling of serum in ischemic stroke patients using UPLC/Q-TOF MS/MS
Source: PLoS One. 2017 Dec 11;12(12):e0189009. doi: 10.1371/journal.pone.0189009 (PMC5724857; doi:10.1371/journal.pone.0189009)

**Figure S1** Trending plot of 12 metabolites in positive ESI mode.

**
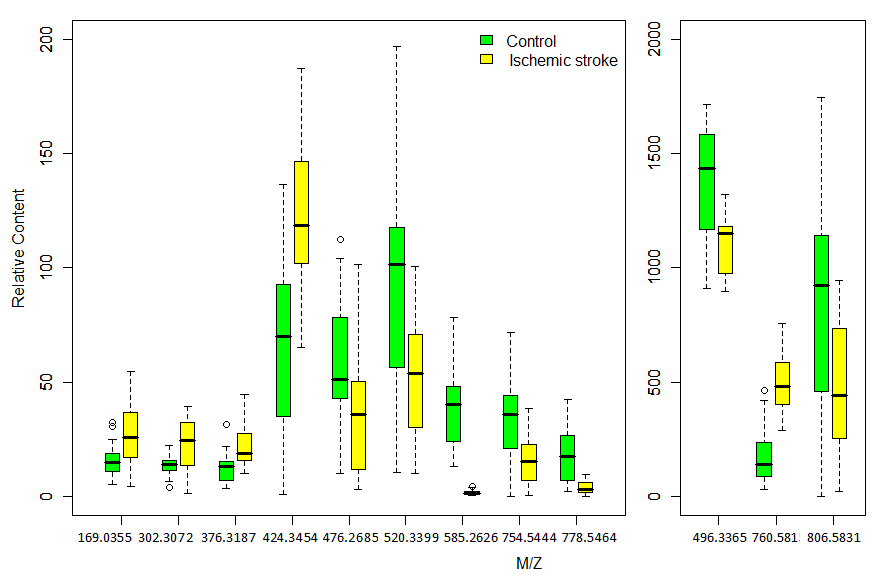
**

Supplement: S1 Fig — (DOC) [file pone.0189009.s002.doc]
