# Supplementary material for: Potential serum biomarkers and metabonomic profiling of serum in ischemic stroke patients using UPLC/Q-TOF MS/MS
Source: PLoS One. 2017 Dec 11;12(12):e0189009. doi: 10.1371/journal.pone.0189009 (PMC5724857; doi:10.1371/journal.pone.0189009)

**Figure S2** Mass fragment information of principal metabolites in positive ion mode. (A) Uric acid; (B) LysoPE(18:3(6Z,9Z,12Z)); (C) LysoPC(18:2(9Z,12Z)); (D) Bilirubin; (E) Sphinganine; (F) Linoelaidyl carnitine; (G) LysoPC(16:0); (H) Adrenoyl ethanolamide; (I) PE(22:1(13Z)); (J) PS(14:1(9Z)); (K) PC(20:4(5Z,8Z,11Z,14Z)); (L) PC(22:6(4Z,7Z,10Z,13Z,16Z,19Z)).


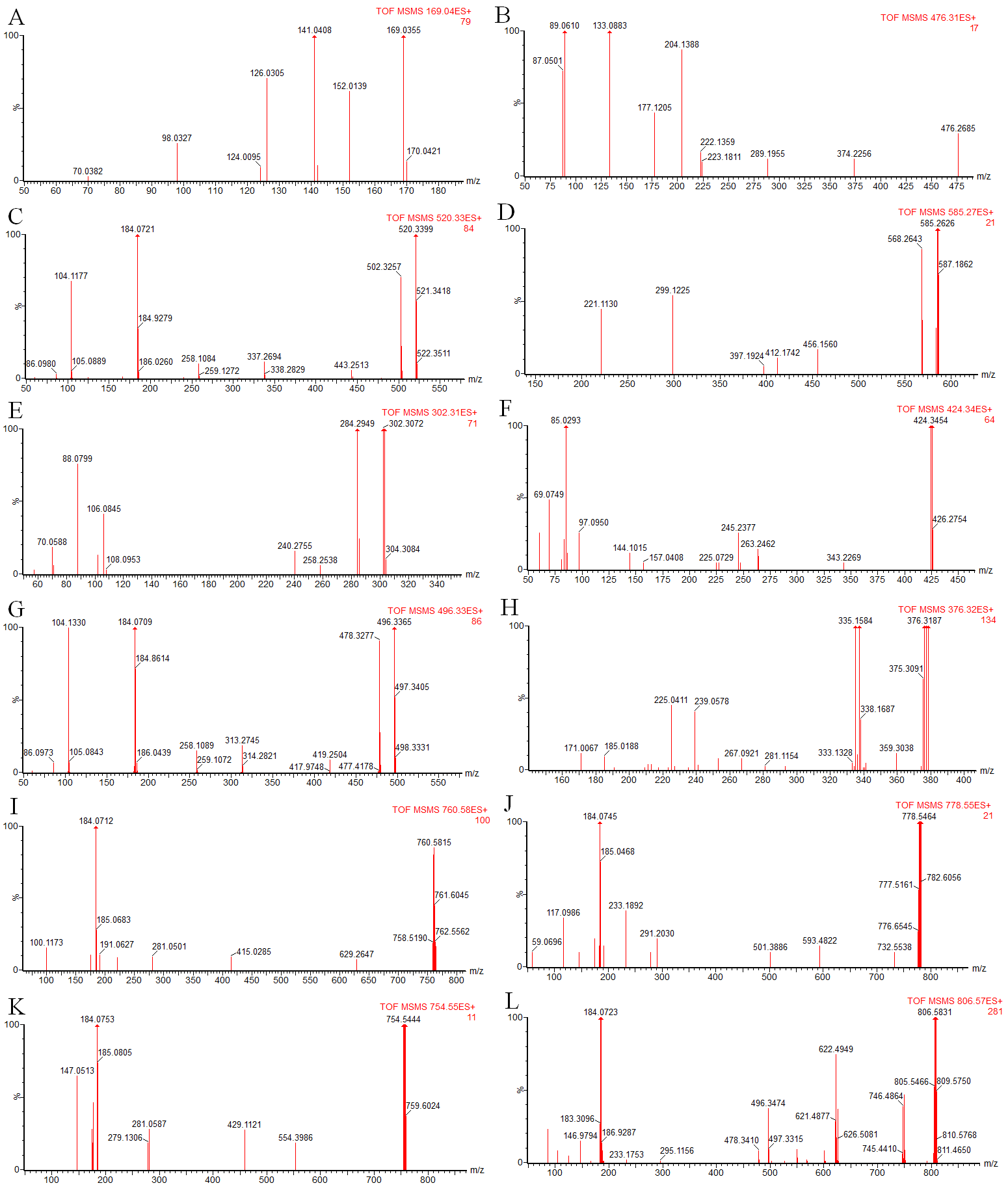

Supplement: S2 Fig — (DOC) [file pone.0189009.s003.doc]
